# Comparison of regional flortaucipir PET with quantitative tau immunohistochemistry in three subjects with Alzheimer’s disease pathology: a clinicopathological study

**DOI:** 10.1186/s13550-020-00653-x

**Published:** 2020-06-15

**Authors:** Michael J. Pontecorvo, C. Dirk Keene, Thomas G. Beach, Thomas J. Montine, Anupa K. Arora, Michael D. Devous, Michael Navitsky, Ian Kennedy, Abhinay D. Joshi, Ming Lu, Geidy E. Serrano, Lucia I. Sue, Anthony J. Intorcia, Shannon E. Rose, Angela Wilson, Leanne Hellstern, Natalie Coleman, Matthew Flitter, Patricia Aldea, Adam S. Fleisher, Mark A. Mintun, Andrew Siderowf

**Affiliations:** 1 Avid Radiopharmaceuticals, 3711 Market St., 7th floor, Philadelphia, PA 19104 USA; 2 Department of Pathology, University of Washington, Seattle, WA USA; 3 Civin Laboratory for Neuropathology, Banner Sun Health Research Institute, Phoenix, AZ USA; 4 Department of Pathology, Stanford University, Stanford, CA USA; 5 Present Address: Medpace Holdings, Inc., Cincinnati, Ohio USA; 6 Present Address: Department of Neurology, University of Pennsylvania, Philadelphia, PA USA

**Keywords:** Flortaucipir, [^18^F]-AV-1451, PET, Alzheimer’s, Tau, NFT, PHF, Autopsy, Histelide

## Abstract

**Background:**

The objective of this study was to make a quantitative comparison of flortaucipir PET retention with pathological tau and β-amyloid across a range of brain regions at autopsy.

**Methods:**

Patients with dementia (two with clinical diagnosis of AD, one undetermined), nearing the end of life, underwent 20-min PET, beginning 80 min after an injection of ~370 mBq flortaucipir [^18^F]. Neocortical, basal ganglia, and limbic tissue samples were obtained bilaterally from 19 regions at autopsy and subject-specific PET regions of interest corresponding to the 19 sampled target tissue regions in each hemisphere were hand drawn on the PET images. SUVr values were calculated for each region using a cerebellar reference region. Abnormally phosphorylated tau (Ptau) and amyloid-β (Aβ) tissue concentrations were measured for each tissue region with an antibody capture assay (Histelide) using AT8 and H31L21 antibodies respectively.

**Results:**

The imaging-to-autopsy interval ranged from 4–29 days. All three subjects had intermediate to high levels of AD neuropathologic change at autopsy. Mean cortical SUVr averaged across all three subjects correlated significantly with the Ptau immunoassay (Pearson *r* = 0.81; *p* < 0.0001). When Ptau and Aβ_1-42_ were both included in the model, the Ptau correlation with flortaucipir SUVr was preserved but there was no correlation of Aβ_1-42_ with flortaucipir. There was also a modest correlation between limbic (hippocampal/entorhinal and amygdala) flortaucipir SUVr and Ptau (Pearson *r* = 0.52; *p* < 0.080). There was no significant correlation between SUVr and Ptau in basal ganglia.

**Conclusions:**

The results of this pilot study support a quantitative relationship between cortical flortaucipir SUVr values and quantitative measures of Ptau at autopsy. Additional research including more cases is needed to confirm the generalizability of these results.

Trial registration, NIH Clinicaltrials.gov NCT # 02516046. Registered August 27, 2015. https://clinicaltrials.gov/ct2/show/NCT02516046?term=02516046&draw=2&rank=1

## Background

The neuropathological hallmarks of Alzheimer’s disease (AD) are extracellular accumulation of aggregated amyloid-β (Aβ) peptides (plaques), often associated with degenerating neurites (neuritic plaques), and neurofibrillary degeneration characterized by intraneuronal aggregates of abnormally phosphorylated microtubule-associated tau protein (Ptau) in paired helical filaments (PHF), historically termed neurofibrillary tangles (NFTs). In autopsy-verified disease, widespread deposition of tau, consistent with high Braak stage, is typically associated with cognitive impairment and is rarely seen in the absence of moderate to frequent neuritic plaques [1–6]. Based on these results, it has been speculated that Aβ and Ptau changes may begin independently [4, 7], but Aβ plaques may be a necessary condition for the neocortical spread of NFTs, and consequent neuronal damage and cognitive impairment in AD [3].

Positron emission tomography (PET) imaging agents targeting Aβ and Ptau have facilitated the exploration of these relationships in living persons. Florbetapir [^18^F], florbetaben [^18^F], and flutemetamol [^18^F] are now approved for use in PET imaging of cerebral amyloid based on studies showing high sensitivity and specificity for predicting moderate to frequent neuritic plaques at autopsy [8–10].

More recently, tracers targeting pathologic tau have been reported [11–20]. The topographical pattern of tau tracer retention on PET imaging appears to roughly follow the neuropathological (autopsy) pattern of NFT accumulation [21], in that tau PET tracer retention is usually limited to mesial temporal lobe in clinically normal subjects, but appears to involve the lateral temporal, parietal and frontal lobes in amyloid-positive subjects with increasing degrees of cognitive impairment [22–28]. Despite these encouraging initial results, it remains to be demonstrated that the pattern of abnormal tau signal on PET imaging of individual AD subjects matches the pattern of Ptau deposition at autopsy. To date, the most well-studied agent has been flortaucipir (flortaucipir [^18^F], also known as flortaucipir F 18, [^18^F]-AV-1451 and [^18^F]-T807), but published imaging-to-autopsy studies with flortaucipir have predominantly evaluated non-AD dementias [29–35]. As expected from the established neuropathology of these conditions, cortical flortaucipir PET signal in these subjects has generally been lower than what would be expected from typical AD patients [33, 35] and, where present, has tended to be greatest in anterior temporal lobes, frontal lobe, and striatum/globus pallidus [30, 31, 33, 34], as well as white matter [30]. Pathological evaluation ranged from semiquantitative scoring to quantitative immunohistochemistry (% area occupied by hyperphosphorylated tau by Ptau staining) and the correlation between flortaucipir PET signal and the estimate of NFT/Ptau burden at autopsy ranged from not significant [30, 31, 34] to excellent (*r* = 0.77–0.93) [29, 32, 33]. In contrast, a strong correlation between flortaucipir SUVr and tissue AT8 staining was recently reported for a patient with autosomal dominant (PSEN1) AD [36].

The objective of this study was to make a quantitative comparison of premortem flortaucipir PET retention and Ptau observed at autopsy, across multiple brain regions with varying densities of pathological tau, in autopsy-verified AD patients, using a robustly quantitative approach to determine regional protein concentration (Histelide technique) [37].

## Methods

### Study design

Two clinically diagnosed AD patients and one patient with dementia of unknown origin, near their end-of-life, were recruited for this pilot phase of a pivotal clinical trial (NCT # 02516046). Participants had an injection of ~370 mBq flortaucipir [^18^F] followed by PET imaging from 80–100 min after injection. After death, bilaterally symmetrical neocortical and limbic blocks of tissue were dissected from 19 limbic and neocortical regions as described below. Subject-specific PET regions of interest corresponding to pathology blocks were constructed for the 19 target regions above as well as the cerebellar cortex in each hemisphere. Regions were hand drawn on the PET images using dimension-scaled photographs of the gross pathology blocks as reference. Standard uptake value ratios (SUVr) were calculated using a cerebellar reference region. Ptau and Aβ were assessed with a quantitative antibody capture assay, using the AT8 and Aβ_1-42_ antibodies, respectively. Regional quantitative estimates of flortaucipir PET signal and Ptau density were compared using regression/correlation statistics.

This protocol was approved by the relevant institutional review boards and all subjects or authorized representatives signed informed consent prior to the conduct of study procedures. This trial was conducted in compliance with the Declaration of Helsinki and the International Conference on Harmonization guidelines on good clinical practice.

### Imaging acquisition and analysis

For flortaucipir PET imaging, subjects received an IV infusion of approximately 370 MBq (10 mCi) of flortaucipir [^18^F], followed approximately 80 min post-dose, by a 20-min brain scan, acquired as four 5-min frames. All PET data were reconstructed with an iterative or row-action maximum likelihood algorithm with a 128 × 128 or 200 × 200 image matrix, pixel size of 2–2.67 mm × 2–2.67 mm, slice thickness of 2–4.25 mm, and post-reconstruction Gaussian filter of 3–5 mm or a relaxation parameter of normal or sharp filter.

Two experienced readers visually interpreted flortaucipir images as having either an AD pattern (τAD: involving, in moderate cases, increased neocortical flortaucipir activity in posterior lateral temporal lobe or, in advanced cases, in the parietal/precuneus regions or in the frontal region in addition to posterior-lateral temporal, parietal, or occipital) or not-AD pattern (τAD−: No increased neocortical activity, or increased neocortical activity isolated to the mesial temporal, anterolateral temporal, and/or frontal regions).

For the quantitative analysis of flortaucipir PET data, the 5-min PET images were summed into a single 20-min image and regions of interest (ROI) were hand drawn by an experienced reader in native space to match the regions used for histopathological sampling (below). Matching was done by comparing x, y, and z axis dimensions as displayed on the photographed brain sections and scaling the location of sampled sections to the PET dimensions. Once scaled, each ROI was drawn to match as closely as possible the anatomic section location (see example in Supplemental Figure 1). Flortaucipir PET signal was expressed as SUVr values at the regional level, by dividing the PET signal in the target area by the average signal within the cerebellar cortex.

### Neuropathology

All neuropathological protocols were performed with appropriate consent and in accordance with applicable regulations and Institutional Review Boards. Brains of subjects were collected at the respective study sites, fixed in 10% neutral buffered formalin for approximately 3 weeks, and shipped to the central study site for processing. Once fixed, brains were coronally sectioned into approximately 1 cm thick slices and samples taken for paraffin processing and embedding using standard cassettes. Tissue samples were processed and embedded in paraffin wax according to standard protocols, sectioned at 5 μm thickness, and mounted on standard charged glass slides for histology or Histelide assays. Levels of Alzheimer’s disease neuropathological change (ADNC), including Braak neurofibrillary stages, were assigned according to National Institute on Aging—Alzheimer’s Association consensus guidelines [38, 39], using immunohistochemical methods for Ptau (AT8 antibody: MN1020, ThermoFisher Scientific) and Aβ (6E10, Covance) pathology, as well as the Bielschowsky silver method for neuritic plaques. As part of the standard neuropathology evaluation, slides were also assessed on a regional basis for the presence of glial or astrocytic (atypical) tau pathology.

Tissue samples/slides prepared for the Histelide analysis included the all the neocortical and limbic regions from each hemisphere required for Braak staging as part of a standard AD neuropathologic diagnosis, specifically hippocampus/entorhinal cortex, amygdala, middle frontal gyrus, superior and middle temporal gyrus, inferior parietal lobe, and Brodmann’s area (BA) 17 and BA 18 in the occipital lobe. Additional regions sampled bilaterally included basal ganglia at the level of the anterior commissure/nucleus basalis, thalamus, anterior cingulate, posterior temporal lobe BA 37, inferior lateral temporal (BA 20, 21), parietal occipital junction (BA 39), precuneus (BA 7), frontal premotor (BA 6), anterior frontal (BA 9), orbital frontal (BA 11), primary motor cortex (BA 4), and basal ganglia at the level of the full development of the lentiform nucleus (caudate, putamen, and globus pallidus).

### Histelide quantitative antibody capture assay

The Histelide assay was performed as previously described [37]. Briefly, formalin-fixed paraffin-embedded brain tissue from study participants was mounted on charged microscope slides at 5 μm thick then deparaffinized and rehydrated using xylenes and isopropanol. Tissue slides were incubated in the primary antibody for 2 h at room temperature. AT8 (MN1020, ThermoFisher Scientific) and H31L21 Aβ (700254, ThermoFisher Scientific) primary antibodies were used to assess Ptau and amyloid pathology, respectively. Primary antibody exposure was followed by alkaline-phosphatase conjugated secondary antibody incubation overnight at room temperature. Tissue slides were incubated in p-nitrophenyl phosphate (pNPP) solution for 90 min and the absorbance of p-nitrophenyl (pNP), the chromogenic product of (pNPP)-alkaline phosphatase reaction, was measured by spectrophotometer. Analytical grade pNP dilution standards were used to create a 4-parameter equation calibration curve (fitted using Spectromax M2 software) to convert absorbance readings to pNP (μg), a surrogate measure of the antibody-bound protein-of-interest in the sample. pNP mass was normalized to total tissue area (i.e., μg/cm^2^), measured using a light microscope and Stereo Investigator software (MBF Bioscience). Non-specific background was subtracted using adjacent tissue slide incubated with IgG isotype control primary antibody, and three internal standard controls were included across all runs to control for batch effects. Additional details are found in the supplemental material.

### Statistical methods

Demographics and subjects’ characteristics, including clinical diagnosis and cognitive assessment by Mini-Mental State Examination (MMSE) were collected. Considering the small sample size, and the exploratory nature of this analysis, a simple Pearson’s correlation analysis was applied to investigate the relationship between pathological and imaging findings. To further explore the potential clustering effect by subject, a partial correlation was run by conditioning on the subject. An additional partial correlation analysis was run by conditioning both by subject and the other pathological measurement. For example, when investigating the relationship between flortaucipir SUVr and quantitative ptau immunohistochemistry, the correlation analysis was adjusted for both subject and amyloid assessment.

## Results

**Fig. 1:**
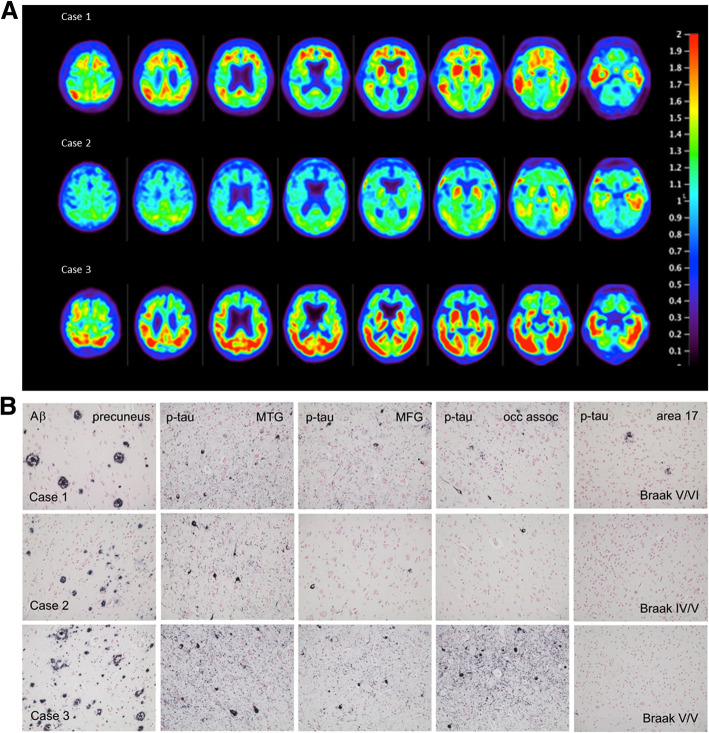
**a** Flortaucipir PET images: premortem evaluation of tau. Flortaucipir PET images acquired 80–100 min post ~370 MBq iv administration. Voxel-wise SUVr values are represented relative to a cerebellar reference region and scaled for a range of 0–2.0. **b** Representative slides showing amyloid (6E10 antibody) and regional Ptau (AT8, MN1020 antibody) pathology in the three cases. MTG: middle temporal gyrus; MFG: middle frontal gyrus; occ assoc: occipital lobe peristriate association cortex, Brodmann area 18

Three participants with a clinical diagnosis of dementia (two clinically diagnosed AD patients and one patient with dementia of unknown origin), with an anticipated life expectancy of less than 6 months at entry, were included in the pilot phase of the study. The intervals between imaging and death for the three cases were 4, 10, and 29 days. Patient characteristics are provided in Table 1. The flortaucipir PET images demonstrating antemortem evidence of pathologic tau in each patient are shown in Fig. 1a. The three subjects varied in degree and distribution of flortaucipir retention on the PET scans. Case 2 showed the least retention, limited primarily to the mesial and lateral temporal lobe. Case 3 showed the most intense PET signal encompassing virtually all of the lateral temporal, occipital, and parietal lobes but with largely right-lateralized activity in the frontal lobe. Case 1 did not have as intense a PET signal as Case 3, but flortaucipir retention was more evident in the frontal lobes, as well as in lateral temporal and parietal lobes. All three cases were visually interpreted as having an AD pattern of pathologic tau (τAD) on flortaucipir PET.

**Table 1:** Characteristics of patients followed to autopsy.

	Case 1	Case 2	Case 3
**Age**	77	86	86
**Gender**	Male	Female	Male
**Race**	White	White	White
**Years of Symptoms**	4	2	8
**MMSE**	NA	20	NA
**Clinical diagnosis**	AD	Dementia Unknown Origin	AD
**Scan to autopsy**^**1**^**interval (days)**	4	10	29
**Death to autopsy interval (days)**	0	0	2
**Thal stage**	4	3	4
**Braak stage (R,L)**	VI/V	IV/V	V/V
**CERAD neuritic plaques**	Frequent	Moderate	Frequent
**Atypical tau pathology**	Absent	Absent	Absent

*Abbreviations*: *AD* Alzheimer’s dementia, *L* left, *NA* not assessed, *R* right

^1^Date of autopsy defined as the start of the autopsy, i.e., date of brain tissue collection

At consensus neuropathological diagnosis (38, 39), Braak stage by hemisphere ranged from IV to VI, but each subject was at least Braak V (B3) in at least one hemisphere. Case 1 had the highest Braak stage score (R/L:VI/V) and Case 2 the lowest (R/L:IV/V). Representative AT8 stained slides are shown in Fig. 1b. All three patients had moderate to frequent CERAD neuritic plaque densities. Cases 1 and 3 had an amyloid Thal phase of 4 (A3), and thus met the criteria for high AD neuropathological change [38]. Thal phase in Case 2 was 3; thus, this case met the criteria for intermediate AD neuropathological change. Atypical Ptau pathology (e.g., glial or morphologically non-AD neuronal tau) was not noted for any subject.

**Fig. 2:**
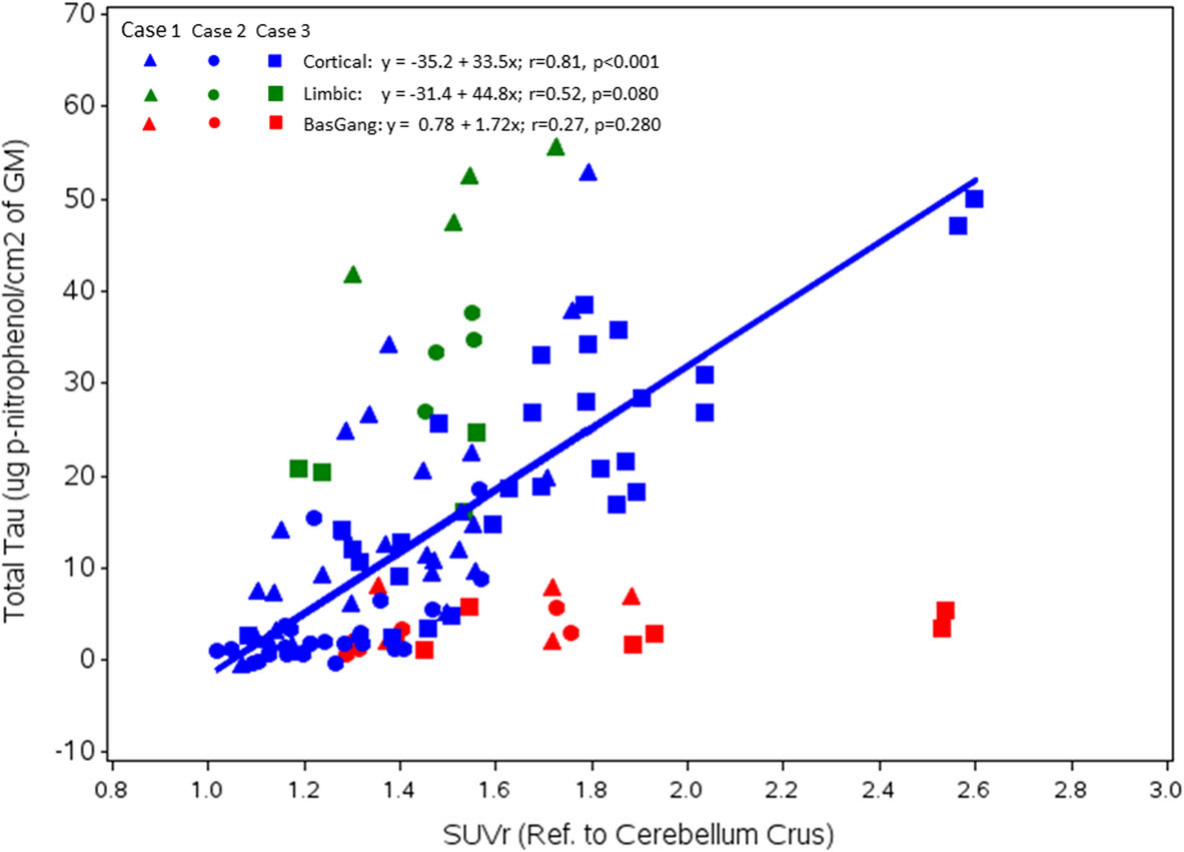
Associations shown between regional flortaucipir SUVr and Ptau total tissue concentration (Histelide) values at autopsy. Regression line shows the relationship between cortical regional SUVr and cortical regional Ptau. Cortical regions are shown in blue, limbic in green, and basal ganglia in red. Symbols differentiate individual subjects

There was a highly significant correlation across all patients between neocortical regional flortaucipir SUVr and the Histelide estimate of Ptau total tissue concentration (Fig. 2, blue symbols; Pearson *r* = 0.81; *p* < 0.0001; Table 2, Model 1). Although the range of values differed across individuals, similar trends were seen within individual subjects (Supplemental Figure 2). Correcting the regression model for individual cases did not meaningfully alter the *r*-value (Pearson *r* = 0.81; *p* < 0.0001; Table 2, Model 2). There was also a modest correlation between limbic (hippocampal/entorhinal and amygdala) flortaucipir SUVr and the Histelide estimate of Ptau total tissue concentration (Fig. 2, green symbols; Pearson *r* = 0.52; *p* = 0.080). However, the slope of the SUVr/Ptau relationship appeared perhaps steeper for these limbic regions than for the cortical regions, suggesting a possibly reduced sensitivity of flortaucipir SUVr to Ptau in these limbic regions. There was no correlation between flortaucipir SUVr and the Histelide estimates for subcortical regions including thalamus, ventromedial globus pallidus, and caudate-putamen (Fig. 2, red symbols; Pearson *r* = 0.27; *p* = 0.280).

**Table 2:** Correlations among cortical flortaucipir SUVr and Histelide estimates of Ptau and Aβ tissue concentrations at autopsy

	Model 1 (no adjustment)		Model 2 (adjusted for case)		Model 3 (adjusted for case and other pathology	
	Pearson ***r***	***p*** Value	Pearson ***r***	***p*** Value	Pearson ***r***	***p*** Value
**Ptau vs SUVr**	0.81	*p* < 0.0001	0.81	*p* < 0.0001	0.73	*p* < 0.0001
**Aβ vs SUVr**	0.61	*p* < 0.0001	0.52	*p* < 0.0001	0.01	*p* = 0.9302
**Ptau vs Aβ**	0.66	*p* < 0.0001				

Model 1: No adjustment; Model 2: partial correlation by adjusting for case ID; Model 3: partial correlation by adjusting for case ID, and the other pathology. For example, the correlation between PHF tau and SUVr was adjusted for case ID and Aβ_1-42_

Table 2 shows that there was also an apparent correlation between neocortical flortaucipir SUVr and the Histelide estimate of Aβ total tissue concentration (Table 2, row 2: Pearson *r* = 0.61; *p* < 0.001). However, this is likely due to colinearity between Ptau and Aβ (Pearson *r* = 0.66; *p* < 0.0001), as significance for the flortaucipir SUVr- Aβ correlation did not survive adjustment for Ptau tissue concentration in multivariable models (Table 2, Model 3, Pearson *r* = 0.01, *p* = 0.9302). In contrast, the correlation between flortaucipir PET SUVr and Histelide estimate of Ptau total tissue concentration survived correction when Aβ was included in the model (Pearson *r* = 0.73, *p* < 0.0001).

## Discussion

The results showed significant associations between neocortical flortaucipir PET signal (SUVr values) and Ptau tissue concentrations across regions among subjects with different Braak stages, and importantly, within subjects but across different brain regions. Our findings are consistent with the widely accepted descriptions of the density and extent of neurofibrillary degeneration in AD in the medical literature [4, 21, 38–40] and provide initial evidence that flortaucipir PET may be an accurate measure of quantitative differences in the density of the Ptau that is most consistently associated with severity of cognitive impairment in AD [4–6].

There was also a correlation between limbic (hippocampal/entorhinal and amygdala) flortaucipir SUVr and the Histelide estimate of Ptau tissue concentration. However, the slope of the relationship appeared steeper than for neocortical measures, suggesting a possibly reduced sensitivity of flortaucipir SUVr to Ptau in these limbic regions. It is not clear whether this is due to artifact of PET signal loss subsequent to atrophy and the resultant partial volume effects on the PET scans [24] or whether this reflects a lower affinity of flortaucipir for the types of pathologic tau found in the mesial temporal lobe of patients with AD [41].

In contrast to neocortical and limbic areas, there was no correlation between flortaucipir PET signal and Histelide estimate of Ptau in the subcortical regions examined (striatum, globus pallidus, nucleus basalis, and thalamus). The lack of correlation of flortaucipir signal with striatum Ptau is consistent with previous studies that have shown presumably “off-target” flortaucipir PET signal in these regions even in cognitively normal elderly subjects that would be expected to have little or no Ptau [22, 26, 42]. The differing kinetics of flortaucipir PET in these regions [42] suggests the presence of a different, lower affinity, high-density binding site that is probably not related to NFTs.

Several clinicopathological case reports have investigated the relationship between flortaucipir imaging and pathological changes in patients with non-AD tauopathies or amyloidoses. One such study showed no selective flortaucipir retention (minimal retention similar to cognitively normal controls) and no increased occurrence of NFTs in subjects with autopsy-confirmed Creutzfeldt-Jakob disease [35]. In a patient with autopsy-confirmed corticobasal degeneration (CBD), the greatest densities of pathological tau and the highest PET SUVrs were found, as might be predicted, in subcortical regions including the putamen and pallidum [29]. Another study, again in a patient with confirmed CBD, showed similar results [32]. A fourth case report, in a patient with autopsy-confirmed Parkinson’s disease, found elevated flortaucipir PET signal in areas previously associated with off-target flortaucipir retention (basal ganglia, midbrain, choroid plexus) and in the inferior temporal cortex, the latter consistent with the evidence of AD-like, possibly age-related, NFT accumulation in the entorhinal cortex at autopsy [31]. The same group found similar results in two progressive supranuclear palsy (PSP) patients and a MAPT P301L carrier [30], with the greatest retention observed on flortaucipir PET in basal ganglion and midbrain, and only low levels of retention in cortical areas; mainly frontal, anterior temporal, and white matter regions. At autopsy, Ptau immunoreactive inclusions in these cases were more widespread and abundant than predicted by the imaging results. However, with the exception of the entorhinal cortex, the Ptau pathology in such subjects is morphologically and biochemically different (e.g., differences in the relative amounts of 3R and 4R tau) from that seen in AD. In contrast, a series of 3 patients with frontotemporal dementia due to a MAPT R406W mutation (which produces 3R/4R tau aggregates similar to those in AD) showed highly significant correlations between flortaucipir PET and neuropathological grading and semi-quantitative assessment of tau-positive neurites [33]. Finally, a case report of a living patient with familial frontotemporal dementia due to a MAPT 10 + 16C>T gene mutation showed a significant association between tau imaging and the familial pattern of pathology [43]. One interpretation of this conflicting data is that there is the heterogeneity of flortaucipir binding in non-AD tauopathies and that flortaucipir is more likely to recognize pathology from patients with MAPT mutations that produce more AD-like tau pathology.

Consistent with this hypothesis, a strong correlation between regional flortaucipir SUVr and percent area labeled by AT8 at autopsy was reported for an autosomal dominant PSEN-1 AD subject. Although flortaucipir SUVr values in the present sporadic AD patients were considerably lower than the SUVr in the autosomal dominant case, a similar relationship was observed in the present study between regional SUVr and regional Ptau, as estimated from AT8-Histelide. Thus, our results contribute to existing evidence of a concordance between imaging and AD pathological change.

We also found a modest correlation between the density of Aβ and Ptau, as separately measured by Histelide, consistent with a previous report [44]. This colinearity was most likely responsible for the apparent correlation between flortaucipir PET SUVr and Aβ_1-42_ tissue concentrations at autopsy. When Ptau was included in the model, the significance of its correlation with flortaucipir SUVr was preserved while that with Aβ_1-42_ was not.

Hyperphosphorylated tau pathology may occur in both glial cells and neurons in both AD and non-AD dementias. In this respect, it is important to note both that the subjects in this study all had sufficient amyloid and Ptau pathology to meet the National Institute of Aging—Alzheimer’s Association criteria for intermediate or high ADNC [38]. However, abnormal tau pathology (e.g., glial tau) was not identified in these cases at autopsy. Thus, it seems unlikely that non-AD glial or neuronal tau pathology is accounting for the observed flortaucipir PET signal.

Alternatively, it has been suggested [45, 46] that flortaucipir binds to monoamine oxidase (MAO). However, it is not clear that the reported in vitro affinity of flortaucipir to MAO translates into in vivo signal. The pattern of retention of flortaucipir does not correspond to the known distribution of MAO activity in the brain (e.g., in contrast to MAO there is limited flortaucipir retention in the thalamus of normal subjects). Recently, it was demonstrated that the AD-tau-like cortical retention for another putative tau tracer, THK 5351, could be displaced by the administration of the MAO-B inhibitor selegeline [47]. It was suggested that rather than, or in addition to tau, THK 5351 might be binding to MAO-B in neuritic plaque-associated astrocytes, thus contributing both to the AD-tau-like pattern of retention. Studies in our lab do not support a high-affinity binding of flortaucipir to MAO-B [48], and patients taking MAO-B blockers do not show reduced retention of flortaucipir [49]. Thus, we believe the most parsimonious explanation for the association between flortaucipir PET and Ptau at autopsy is that flortaucipir binds to and reflects the density of Ptau in the brain, except for off-target regions where the basis of the binding remains unknown.

Our results must be considered in the context of some study limitations. Because of the fragile condition of the subjects (all within 30 days of death), MRI was not performed, which limits the precision with which the PET ROI can be matched to the histopathology blocks. Additionally, the Histelide values were derived from slides representing only a small portion of that block, whereas the PET ROIs, albeit drawn to match the histopathology blocks, represent a much larger area than an individual slide. SUVr values were calculated using a cerebellum reference region. The pathology expert panel did not evaluate NFT in the cerebellum, but Histelide estimates of phosphorylated protein levels in the cerebellum were less than or equal to estimates in other presumably low tau subcortical regions. Perhaps most important, this is a small case-series and only included subjects with clinically defined dementia, with autopsy evidence of intermediate to high ADNC (all three cases had at least Braak V NFT stage, and moderate to frequent neuritic plaques but Case 2 only had a Thal amyloid phase of 3). The range of flortaucipir SUVr and Histelide Ptau estimates varied among the three subjects (Supplemental Figure 1). Given the small sample size in this study, it is fair to conclude that additional subjects would be required to more precisely characterize the degree of relationship between flortaucipir PET signal and Ptau density, particularly with respect to limbic regions, where only six data points are available for evaluation.

A strength of the study is that PET regions of interest were drawn and PET scans interpreted without knowledge of quantitative histology results; histology was interpreted in a blinded fashion as well. Although it is possible that other approaches (e.g., mapping of anatomically derived standard regions to the PET scans) could have been taken, the current strategy has the further advantage of closely matching the location of the PET ROIs to the individual regions sampled for histopathological evaluation. Other strengths of this study include the standardized acquisition of PET data and the fact that brain samples came from two different clinical sites.

## Conclusion

In conclusion, this study provides preliminary evidence that flortaucipir PET imaging captures the density and distribution of pathologic neocortical tau in a manner that is correlated with findings from a quantitative antibody capture assay. By extension, these findings suggest that flortaucipir PET signal may be useful in assessing neurofibrillary degeneration in AD.

## Supplementary information

**Additional file 1: Supplemental Figure 1.** Example of PET ROI hand drawn to match pathology sample block, in this case right amygdala. Note the PET image is in radiologic orientation so the relevant ROI for this example is on the left of the image (blue). **Supplemental Figure 2.** Correlation between regional flortaucipir SUVr and Histelide estimate of regional Ptau in individual subjects. Blue symbols, regression line and Pearson’s r are for cortical regions. Green symbols reflect limbic regions: hippocampus/entorhinal cortex and amygdala. Red symbols represent subcortical regions: caudate/putamen, basal ganglia/nucleus basalis, thalamus. **Supplementary Table.**

## Data Availability

Flortaucipir SUVr and Ptau values shown in Fig. 2 and the supplemental figure are included in the supplemental materials.

## Abbreviations

AD: Alzheimer’s disease
ADNC: Alzheimer’s disease neuropathological change
Aβ: Amyloid β
CBD: Corticobasal degeneration (corticobasal syndrome)
BA: Brodmann area
MAO: Monoamine oxidase
MMSE: Mini-Mental State Examination
MRI: Magnetic resonance imaging
NFT: Neurofibrillary tangle
PET: Positron emission tomography
PHF: Paired helical filament tau
pNP: p-Nitrophenol
pNPP: p-Nitrophenol phosphate
PSP: Progressive supranuclear palsy
Ptau: Abnormally phosphorylated tau
ROI: Region of interest
SUVr: Standardized uptake value ratio
