# Supplementary material for: Comparison of regional flortaucipir PET with quantitative tau immunohistochemistry in three subjects with Alzheimer’s disease pathology: a clinicopathological study
Source: EJNMMI Res. 2020 Jun 15;10:65. doi: 10.1186/s13550-020-00653-x (PMC7295920; doi:10.1186/s13550-020-00653-x)
Supplement: Supplementary file 1 — Additional file 1: Supplemental Figure 1. Example of PET ROI hand drawn to match pathology sample block, in this case right amygdala. Note the PET image is in radiologic orientation so the relevant ROI for this example is on the left of the image (blue). Supplemental Figure 2. Correlation between regional flortaucipir SUVr and Histelide estimate of regional Ptau in individual subjects. Blue symbols, regression line and Pearson’s r are for cortical regions. Green symbols reflect limbic regions: hippocampus/entorhinal cortex and amygdala. Red symbols represent subcortical regions: caudate/putamen, basal ganglia/nucleus basalis, thalamus. Supplementary Table. [file 13550_2020_653_MOESM1_ESM.zip › 13550_2020_653_MOESM1_ESM.docx]

**Comparison of Regional Flortaucipir PET with Quantitative Tau Immunohistochemistry in Three Subjects with Alzheimer’s Disease Pathology:**

**A Clinico-Pathological Study**

Michael J. Pontecorvo^1^, C. Dirk Keene^2^, Thomas G. Beach^3^, Thomas J. Montine^4^,

Anupa K. Arora^1^, Michael D. Devous Sr^1^, Michael Navitsky^1^, Ian Kennedy^1^,

Abhinay D. Joshi^1,5^, Ming Lu^1^, Geidy E. Serrano^3^, Lucia I. Sue^3^,

Anthony J Intorcia^3^, Shannon E. Rose^2^, Angela Wilson^2^, Leanne Hellstern^2^,

Natalie Coleman^2^, Matthew Flitter^1^, Patricia Aldea^1^

Adam S. Fleisher^1^, Mark A. Mintun^1^, Andrew Siderowf^1,6^

^1^Avid Radiopharmaceuticals, Philadelphia, PA

^2^Department of Pathology, University of Washington, Seattle, WA

^3^Civin Laboratory for Neuropathology, Banner Sun Health Research Institute, Phoenix AZ

^4^ Department of Pathology, Stanford University, Stanford, CA

^5^ Present Address: Medpace Holdings, Inc., Cincinnati, Ohio

^6^Present Address: Department of Neurology, University of Pennsylvania, Philadelphia PA

Corresponding author:

Michael J. Pontecorvo

Avid Radiopharmaceuticals

3711 Market St., 7^th^ floor

Philadelphia, PA, USA 19104

[pontecorvo@avidrp.com](mailto:pontecorvo@avidrp.com)

908-672-2581

**Pontecorvo et al., Supplemental Information Regarding Histelide Assay**

Histelide is an antibody capture assay that uses slides with formalin-fixed paraffin-embedded tissue sections and Beer’s law to quantify results (Postpuna et al., Brain Pathol. 2012 Jul; 22(4): 472–484).  Slides were deparaffinized and rehydrated by standard means: xylene (4 times, 10 min each), followed by 1:1 mixture of xylene and 100% isopropanol (2 times, 5 min each), 100% isopropanol (2 times, 5 min each), 96% isopropanol in water (3 min), 70% isopropanol in water (3 min), 50% isopropanol in water (3 min), followed by two 5 min washes with TBST (10 mM Tris-HCl pH 7.8 + 100 mM NaCl + 0.05% Tween), and then pre-treated with 88% formic acid for 5 min. Slides were washed with TBST 3 times for 10 min, and incubated in blocking solution (5% normal goat serum, 2% bovine serum albumin, 0.25% triton in TBST) overnight at 4°C. Slides were then incubated with primary antibody (Aβ: H31L21 Aβ, 700254, Invitrogen, Camarillo, CA / ThermoFisher Scientific, Waltham, MA, 0.5 µg/ml; Ptau: AT8, MN1020, Invitrogen, Camarillo, CA / Thermofisher Scientific, Waltham, MA, 0.4 µg/ml) for 8 hours at room temperature, washed three times for 10 min each in blocking solution, and then incubated with 1.5 µg/ml alkaline phosphatase–conjugated goat IgG anti-rabbit (Aβ) or anti-mouse (Ptau) secondary antibody (Jackson Immunoresearch Laboratories, West Grove, PA) overnight at room temperature. Incubation with the secondary antibody was followed by three 15 min TBST washes. To measure p-nitrophenyl phosphate (PNPP) absorbance, slides were first washed with diethanolamine (DEA) solution (10 mM diethanolamine and 0.5 mM MgCl2, pH 9.5) for 5 min. incubated with PNPP solution (Sigma-Aldrich, St Louis, MO, or ThermoFisher Scientific, Waltham, MA), for 2–4 hours, depending on the rate of PNPP development. All absorbance data was between 0.5 and 2.5 absorbance units to maximize linearity of signal. Data is normalized to gray matter area in each individual section using a microscope with a motorized stage and StereoInvestigator software. Controls include non-specific IgG primary antibody control sections paired (adjacent) to each study section from each region from each case and positive control sections from cases (3) with high ADNC run in every batch. For each Antibody, 14 runs were performed with up to three brain regions (all cases) per run. Batch effects (calculated from variation in positive controls) ranged from -15% to +25% from index run, and results were normalized accordingly.

**Reference:**

[Postupna N](https://www.ncbi.nlm.nih.gov/pubmed/?term=Postupna%20N%5BAuthor%5D&cauthor=true&cauthor_uid=21999410), [Rose SE](https://www.ncbi.nlm.nih.gov/pubmed/?term=Rose%20SE%5BAuthor%5D&cauthor=true&cauthor_uid=21999410), [Bird TD](https://www.ncbi.nlm.nih.gov/pubmed/?term=Bird%20TD%5BAuthor%5D&cauthor=true&cauthor_uid=21999410), [Gonzalez-Cuyar LF](https://www.ncbi.nlm.nih.gov/pubmed/?term=Gonzalez-Cuyar%20LF%5BAuthor%5D&cauthor=true&cauthor_uid=21999410), [Sonnen JA](https://www.ncbi.nlm.nih.gov/pubmed/?term=Sonnen%20JA%5BAuthor%5D&cauthor=true&cauthor_uid=21999410), [Larson EB](https://www.ncbi.nlm.nih.gov/pubmed/?term=Larson%20EB%5BAuthor%5D&cauthor=true&cauthor_uid=21999410), et al. Novel antibody capture assay for paraffin-embedded tissue detects wide-ranging amyloid beta and paired helical filament-tau accumulation in cognitively normal older adults. [Brain Pathol](https://www.ncbi.nlm.nih.gov/pubmed/?term=postuna+n+AND+rose+se+2012). 2012; 22: 472-484.

**Pontecorvo et al., Supplemental Figures**

**Supplemental Figure 1**. Example of PET ROI hand drawn to match pathology sample block, in this case right amygdala. Note the PET image is in radiologic orientation so the relevant ROI for this example is on the left of the image (blue).


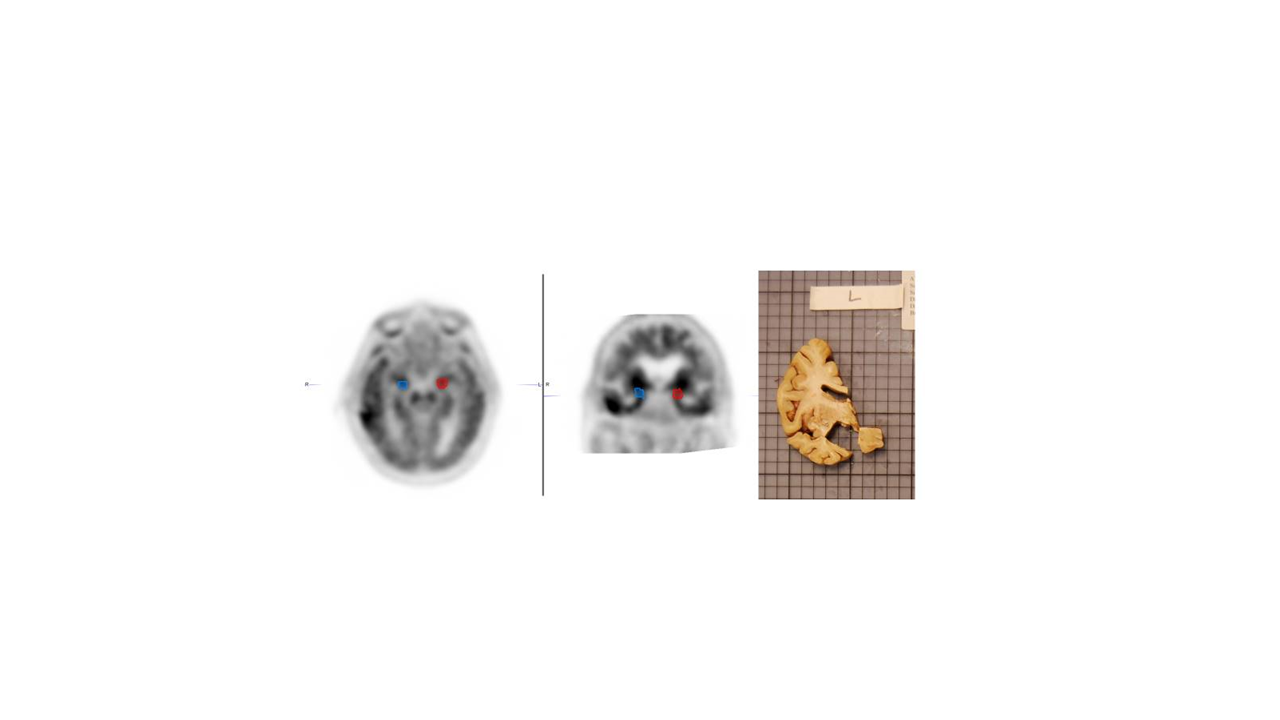


**Supplemental Figure 2.** Correlation between regional flortaucipir SUVr and Histelide estimate of regional Ptau in individual subjects. Blue symbols, regression line and Pearson’s r are for cortical regions. Green symbols reflect limbic regions: hippocampus/entorhinal cortex and amygdala. Red symbols represent subcortical regions: caudate/putamen, basal ganglia/nucleus basalis, thalamus


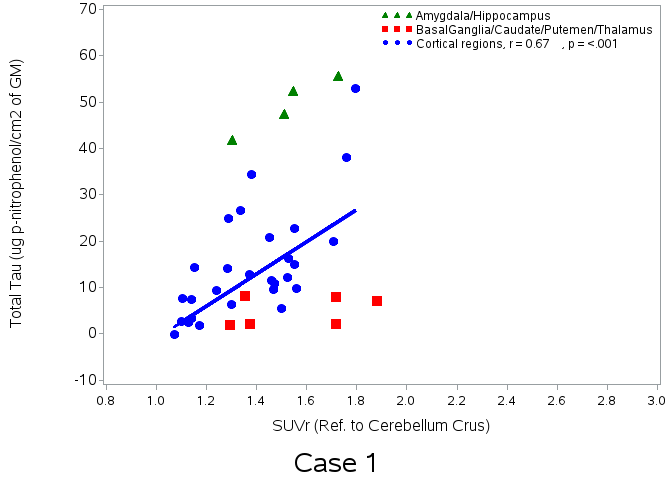

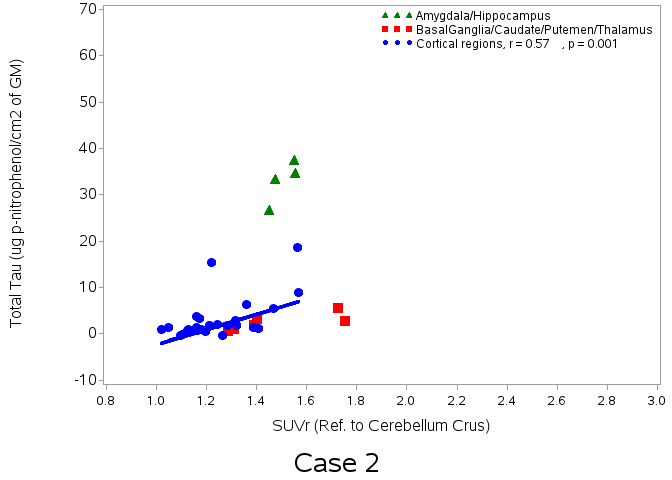

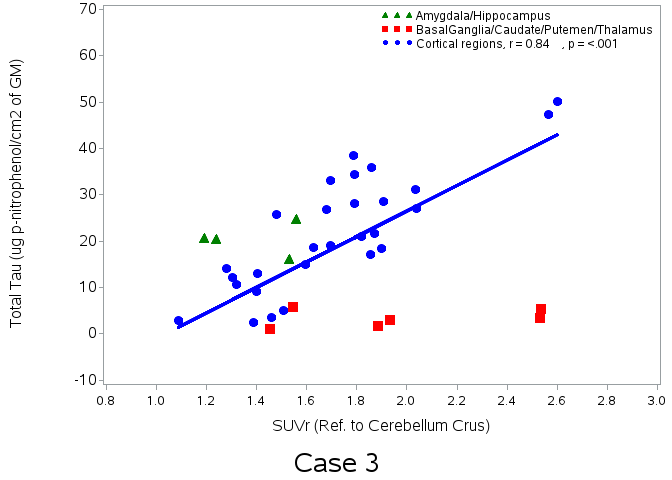


**Pontecorvo et al., Supplemental Table (Excel Spreadsheet Attached)**

Regional SUVr, Ptau and Aβ values for each case in descending order of SUVr
